# Attitudes and Expectations Towards Mental Health Interventions in the General Population: Comparing Face-to-Face Counseling, Blended Counseling, and Digital or On-Paper Self-Help

**DOI:** 10.32872/cpe.16235

**Published:** 2025-11-28

**Authors:** Nele A. J. De Witte, Fien Buelens, Jennifer Apolinário-Hagen, Tom Van Daele

**Affiliations:** 1 Psychology & Technology, Centre of Expertise Care and Well-being, Thomas More University of Applied Sciences, Antwerp, Belgium; 2 Institute for Occupational, Social and Environmental Medicine, Faculty of Medicine, Centre for Health and Society (chs), Heinrich Heine University Düsseldorf, Düsseldorf, Germany; 3 Centre for Technological Innovation, Mental Health and Education, Queen’s University Belfast, Belfast, United Kingdom; Philipps-University of Marburg, Marburg, Germany

**Keywords:** mental healthcare, technology acceptance, self-help, digital mental health, blended care

## Abstract

**Background:**

Digital interventions are supported by a growing evidence base and have the potential to contribute to accessible and personalized mental healthcare services. When individuals seek help for mental health problems, various intervention options are available in a digital, face-to-face or on-paper format. However, it is important to understand what individuals find important for intervention selection and how they perceive different intervention options.

**Method:**

The study recruited 232 individuals for a cross-sectional online survey on (1) the relevance of 12 evaluation dimensions for mental health support, (2) whether self-help books, digital interventions, face-to-face counseling, and blended interventions would meet expectations, and (3) self-reported likelihood of use.

**Results:**

The most important dimensions for intervention selection were helpfulness, personal support, motivates to get better, and credibility. Face-to-face counseling was evaluated favorably for dimensions linked to intervention content (e.g., helpfulness), while self-help approaches were rated more positively for practical aspects (e.g., waiting time). Blended counseling received fairly similar dimension ratings as face-to-face counseling. Self-reported likelihood of use differed significantly between modalities despite large individual differences. Face-to-face interventions were most likely to be used, followed by blended counseling, with digital and on-paper self-help options sharing third place.

**Conclusion:**

The findings suggests that mere self-help (online or on paper) does not sufficiently meet the needs and is not the preferred choice for handling mental health problems for most individuals. If presented with the choice, individuals still prefer face-to-face counseling. Nevertheless, blended interventions can be a promising treatment option for the future.

## Highlights

- Mental health services are diversifying but we need to assess attitudes to intervention options.
- Face-to-face support was perceived to be more helpful, motivational and credible than alternatives.
- Self-help approaches were rated more positively for practical aspects but were not preferred.
- Blended interventions were well received but relying on face-to-face support was deemed more likely.

Mental health problems are one of the leading causes of burden worldwide (GBD 2019 Mental Disorders Collaborators, 2022). However, many people, especially young adults, do not seek or find professional help (Bryant et al., 2022). For example, depression is a common and sever mental health complaint but Moitra et al. (2022) calculated that about 39% of individuals with this disorder in high-income countries do not receive treatment. The study by Mauerhofer et al. (2009) in Switzerland found that only 13% of young adults identified by their GP as experiencing depression or sadness actually sought help for that issue. However, help-seeking can also occur beyond face-to-face mental health services in the realm of self-help. In the past, self-help was mainly limited to books, which have demonstrated a moderate degree of effectiveness (Kavanagh & Proctor, 2011; Marrs, 1995). However, individuals now increasingly come across technological resources for self-help as well.

A review on online help-seeking behavior in young people found that the internet can be a gateway to further mental health information and knowledge, a means of connecting with peers or professionals regarding mental health problems, and an alternative to offline help-seeking behavior (Pretorius et al., 2019). Digital self-help services come in various forms, from an informative app or website to a full online treatment program (De Witte et al., 2021). Digital self-help interventions can be provided in an unguided (fully autonomous) or guided format which includes support from a professional who (a)synchronously monitors progress and/or provides feedback. There is a large body of empirical support from clinical trials for the efficacy of digital self-help, such as internet-based cognitive behavioral therapy for depression (Karyotaki et al., 2021). The meta-analysis of Madrid‐Cagigal et al. (2025) also supported the effectiveness of digital mental health interventions for university students with mental health difficulties. Whether a guided format should be preferred can be dependent on the context and intervention tagets since the study of Madrid‐Cagigal et al. (2025) observed a greater effect size for fully automated interventions on anxiety symptoms but not depressive symptoms (perhaps linked to deficits in intrinsic motivation linked to depressive complaints). Self-help applications can show very high drop-out rates. Retention rates as low as 3.3% have been recorded for frequently installed, unguided mental health apps during a 30-day period “in the wild” (Baumel et al., 2019). Retention rates have also been noted as a challenge for the use of self-help books. While bibliotherapy drop-out rates can be acceptable in controlled research with individuals who have a personal interest in the topic, there is little evidence on book completion rates “in the wild” (0-30%; Gualano et al., 2017; Kavanagh & Proctor, 2011). It is also relevant to assess the reach of self-help interventions, as it has been suggested that, for example, book readership might consist of more highly educated individuals and include many who are merely interested in the topic and are not using it as self-help per se (Bergsma, 2008; Kavanagh & Proctor, 2011).

While low perceived need for treatment proves to be one of the barriers to help-seeking behavior in mental health (even in individuals with severe mental health problems; Andrade et al., 2014), digital mental health services can face additional uptake and drop-out challenges. The uptake of digital mental health can be hindered or facilitated by technology acceptance, i.e., attitudes and expectations of end users towards technology (Philippi et al., 2021; Venkatesh et al., 2003). Relevant predictors in the Unified Theory of Acceptance and Use of Technology (UTAUT) are the extent to which individuals believe that a technology can help them achieve their goals (performance expectancy), the amount of effort required to use a technology (effort expectancy), and the opinions of important others regarding technology use (social influence). In line with the role of performance expectancy, the perceived quality and effectiveness of digital interventions has proven to be a key factor in the acceptance of digital mental health interventions for depression, anxiety and stress in adolescents and young adults (Zhu et al., 2025). Several other barriers for using online interventions have been reported. Examples are difficulties with fitting the intervention into the schedule, the lack of a convenient place to perform the intervention, past experiences with an intervention, perceived credibility, and confidentiality (Borghouts et al., 2021). On the other hand, young people have reported that anonymity, privacy, accessibility, inclusivity, and the ability to connect with others are benefits of online help-seeking, which could motivate people to use digital mental health services (Ho et al., 2025; Pretorius et al., 2019).

Alongside (guided) self-help, digital mental health services can also be used in combination with face-to-face interventions in stepped or matched care approaches in blended care. A blended care model can vary greatly in implemented therapeutic approach, objectives, and digital and face-to-face component features, which poses challenges for defining the concept and evaluating effectiveness (Ehrt-Schäfer et al., 2023; Singh et al., 2022). Reviews have provided initial indications of feasibility and effectiveness when compared to face-to-face interventions in serious mental illness (Cohen et al., 2024; Cooper et al., 2022; Ehrt-Schäfer et al., 2023; Erbe et al., 2017; Köhnen et al., 2021). Cooper et al. (2022) documented significant symptom reduction in blended treatment for anxiety and obsessive-compulsive spectrum disorders, and this reduction was found to be greater than the comparison group in a quarter of the included studies. Erbe et al. (2017) observed lower drop-out and/or greater abstinence as compared to face-to-face treatment in six out of nine studies in patients with substance abuse. While reviews focus on serious mental illness, blended approaches have also been identified as promising for mental health prevention and promotion (Singh et al., 2022). However, it is relevant to note that all reviews identify a need for additional rigorous effectiveness and cost-effectiveness research.

Implementation of digital and blended interventions in practice is hindered by a lack of a clear concept on how technology can be embedded in the healthcare system (including funding challenges) as well as a lack of training regarding digital mental health implementation and differences in the acceptance and preferences regarding digital technology in patients or professionals (Cohen et al., 2024; Titzler et al., 2018). This implies that a limited number of individuals might have already come into contact with digital interventions or blended care. While several reviews and meta-analyses address the acceptance of digital health interventions (e.g., Lau et al., 2024), their scope is often restricted to clinical populations and specific intervention types or indications. Similarly, reviews and models (e.g., UTAUT) exploring the drivers and barriers to adoption predominantly focus on digital interventions in isolation, neglecting comparisons with face-to-face and other modalities like self-help books. Few studies directly evaluate and compare intervention modalities on the same dimensions.

Since individuals have diverse treatment options available, it is relevant to gain insight into the determinants that individuals deem important for mental health interventions and analyze to what extent intervention modalities are expected to fulfill these needs. In 2014, Musiat and colleagues designed a study to develop a list of evaluation dimensions which influenced people’s decision to engage with a particular intervention for mental health problems (Musiat et al., 2014). They subsequently investigated to what extent different interventions fulfilled participant’s needs. Their findings indicated that face-to-face interventions were expected to perform well in terms of helpfulness, personal support, credibility, motivational aspects, suiting learning preferences, including feedback, appeal, and credibility. Books, websites, and apps performed well in terms of convenient location and time, anonymity, and no waiting time but were rated poorly on all other dimensions. This also resulted in the highest likelihood of using face-to-face interventions, followed by self-help books and web-based interventions. Smartphone applications showed the lowest likelihood of use.

The study by Musiat et al. (2014) is one of the few studies incorporating such a differentiated assessment (grounded in service users’ needs) of multiple intervention modalities. However, the position of technology in society has altered and the COVID-19 pandemic has resulted in large number of potential first experiences with digital mental health (Parsons et al., 2023), and a decade later, an update of the work of Musiat et al. (2014) seems warranted. Therefore, the aim of this study was to (1) gain insight into the importance of different evaluation dimensions for mental health interventions, (2) assess to what extent individuals from a general and student population expect different intervention options (self-help books, digital interventions, blended counseling, and face-to-face counseling) to meet their expectations, and (3) compare the self-reported likelihood of use between these intervention modalities (including an exploratory subgroup analysis for existing mental health complaints).

## Method

### Participants

First-year applied psychology students at Thomas More University of Applied Sciences in Belgium (aged 17 or older) were invited for participation in the study through e-mail and received course credits for their participation. Additionally, a call for participation was launched in the general population through social media and e-mail. Recruitment took place in November and December in 2023. The study was approved by the Ethical Committee of Thomas More University of Applied Sciences (ECTP2324_03). All participants provided informed consent.

### Questionnaire

The questionnaire was adapted from Musiat et al. (2014) and was distributed (in Dutch) via a link to an online survey platform QuestionPro (https://www.questionpro.com/). Besides the translation, the questionnaire underwent some alterations based on progression in the field regarding the intervention options. The included intervention options were slightly modified to better represent current digital mental health use. Web-based interventions and smartphone applications were merged into a single intervention option, ‘digital interventions,’ since they are applied in a similar way (in the capacity of (guided) self-help) and the difference between these options has grown smaller due to the rise of mobile website traffic and mobile-first web design. Blended counseling, i.e., combining face-to-face and digital interventions, is a newly added intervention option. Self-help books and face-to-face counseling were retained. The 12 evaluation dimensions, which Musiat et al. (2014) developed based on an advisory group of service users, were retained, although learning style was translated to 'matching one's own learning process' to facilitate comprehension by the participants.

Participants were asked to what extent they found 12 criteria or dimensions important, on a 7-point Likert scale ranging from 1 (totally unimportant) to 7 (very important), when looking for help in the broad context of interventions for common mental health problems such as anxiety or depression. These dimensions relate to both the content of the intervention or practical considerations. Dimensions considered that the intervention (1) helps with the problem, (2) motivates to get better, (3) is credible, (4) is accessible without waiting time, (5) is accessible at an appropriate time, (6) provides feedback, (7) includes personal support, (8) is accessible at a convenient location, (9) is free of charge, (10) is attractive/appeals to me, (11) can be consulted anonymously, (12) connects to my own learning preferences (mainly referring to how individuals prefer to process information). As a next step, they rated to what extent they thought that face-to-face counseling, a self-help book (without additional support), blended counseling, and a digital intervention would meet these criteria (on a scale from 1 to 7). The descriptions of the four intervention options, which were provided to the participants, can be found in Table 1. Participants subsequently rated the likelihood of using these modalities (“How likely is it that you would use the following applications?”) on a 6-point Likert scale ranging from 0 (very unlikely) to 5 (very likely).

**Table 1:** Descriptions of the Four Intervention Modalities

Modality	Description
Face-to-face counseling	Face-to-face counseling is when counseling takes place in person. As a client, you see the counselor in the same physical space. You do not use technological aids in the process.
Self-help book	A self-help book is scientifically based and offers insights into problems or challenges that you are struggling with. A self-help book often includes strategies on how to learn to cope with your symptoms. A self-help book can be used alongside other forms of therapy, or on its own^a^.
Blended counseling	Blended counseling is a counseling service that combines face to face and technological interventions. For example, as a client, you regularly see the health professional physically. In addition, you occasionally have conversations via computer (through online consultations). Between sessions, you can also call on a website or smartphone app for additional support.
Digital intervention	In a digital intervention, you receive information, advice or support through a website, app or online platform. You learn more about complaints or problems, complete a self-test, or carry out assignments. In some digital interventions, you also receive remote support (e.g., a health professional who supports you via chat), but this is not always the case.

^a^Participants were asked to reflect on stand-alone implementation of the self-help book for the ratings.

Participants were asked to report whether they currently or previously faced mental health problems and had experience with one or more of the four intervention options mentioned above. Finally, the questionnaire also asked for demographics (gender, age, educational level) and included a question about the frequency of using the internet and different devices (smartphone, computer or laptop, and tablet) to provide an indication of participants’ familiarity with technology use.

### Analysis

The data was analysed using SPSS 29. Individuals with incomplete questionnaire responses were excluded. The data was non-normally distributed according to visual inspection of the data and Kolmogorov-Smirnov Tests of Normality, so non-parametric tests were used. Related-Samples Friedman's Two-Way Analysis of Variance by Ranks and Dunn’s Pairwise Post Hoc tests were used to compare the importance of the dimensions and the ratings between modalities. An Independent-samples Mann-Witney U test was used to in an exploratory analysis to compare between subgroups. The alpha error probability used to test statistical significance was .05 and *p*-values were adjusted by the Bonferroni correction for multiple tests.

## Results

### Sample

While 354 individuals accessed the questionnaire, a large number of incomplete responses resulted in a total of 232 participants from a general (*n* = 106; 46%) and college student (*n* = 126; 54%) population being included. Table S1 in Supplementary Materials provides an overview of the demographics. Apart from age and education, the subsamples differed in tablet usage and gender. Smartphone, internet, and computer or laptop use were high in the sample (ranging from regularly to very often). Despite this being a general population sample, 65.5% of respondents reported current or past mental health problems. However, almost none had experience with any of the four intervention modalities. One participant had experience with face-to-face counseling, and two others had used a self-help book. No experience with digital or blended help was reported.

### Importance of the 12 Dimensions

Table 2 provides an overview of the median and mean importance rating assigned to the different dimensions. All dimensions were deemed important, apart from perceived costs (‘free of charge’), which received a more neutral score. Related-Samples Friedman’s Two-Way Analysis of Variance by Ranks (χ^2^(11) = 610.20, *p* < .001) showed significant differences between the importance of the dimensions. Helpfulness, personal support, motivation, and credibility were deemed the most crucial, with their importance differing significantly from other dimensions, but not among these four. On the other hand, being free of charge was rated significantly least important of all the dimensions. Full results of the Dunn’s Pairwise Post Hoc tests for each dimension can be found in Table S2 in Supplementary Materials.

**Table 2:** Subjective Importance Ratings for the 12 Dimensions

Dimension	Importance	
	*Mdn*	*M* (*SD*)
Helps with the problem	7	6.43 (0.84)
Includes personal support	6	6.24 (0.84)
Motivates to get better	6	6.20 (1.01)
Is credible	6	6.12 (1.01)
Is accessible without waiting	6	5.87 (1.03)
Is accessible at an appropriate time	6	5.84 (1.03)
Provides feedback	6	5.61 (1.05)
Is accessible at a convenient location	6	5.53 (1.12)
Connects to my own learning	6	5.47 (1.12)
Can be consulted anonymously	5	5.15 (1.61)
Is attractive/appeals to me	5	5.11 (1.33)
Is free of charge	4	4.53 (1.37)

*Note.* Scale from 1 (totally unimportant) to 7 (very important).

### Expectations Regarding the Four Modalities

**Figure 1:**
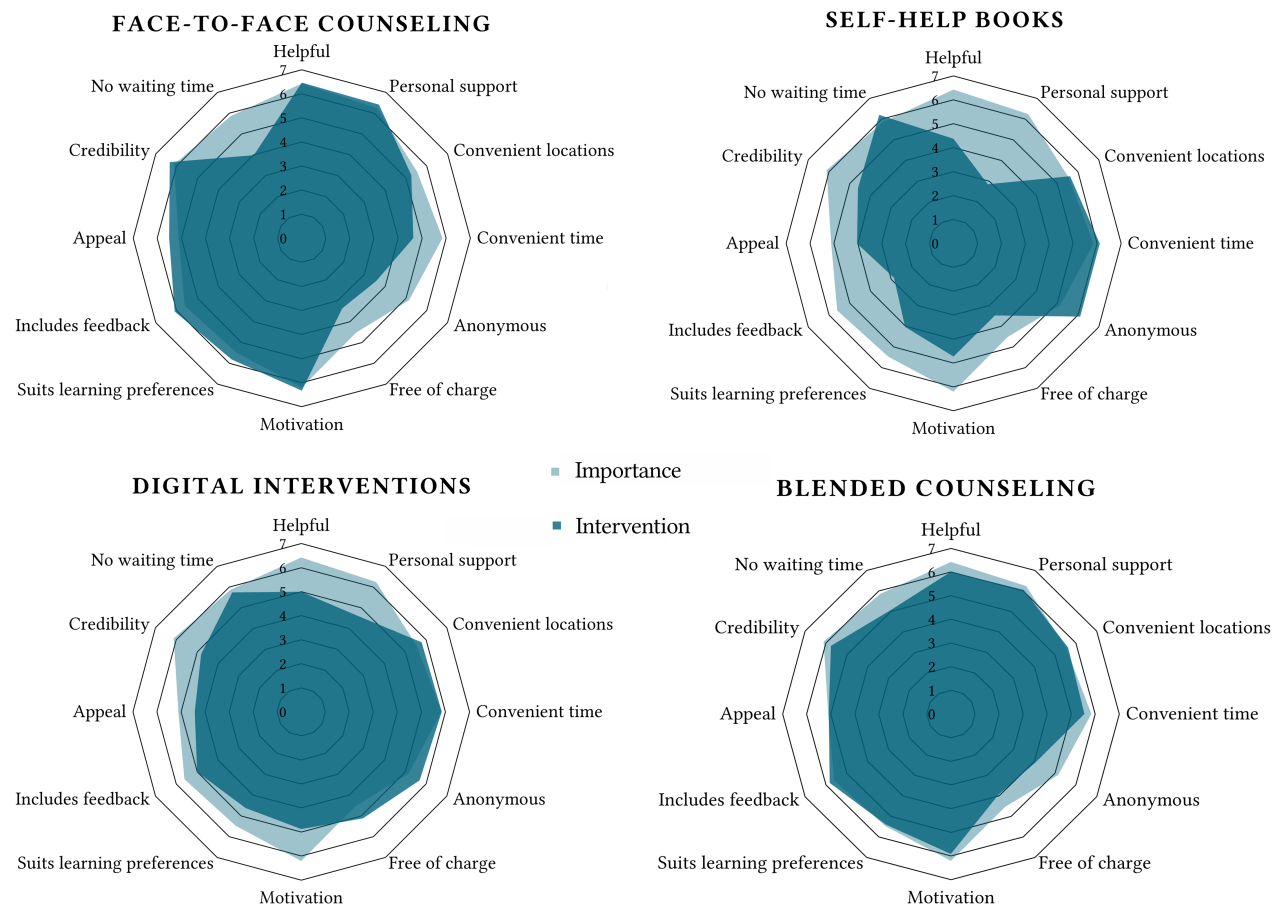
Spider Diagrams Combining Importance and Expectations Ratings for the Four Modalities

Participants rated the four different intervention modalities on the 12 dimensions. Table 3 provides the scores and Figure 1 provides a visual overview of results. Significant differences in terms of expectations across the intervention options were reported on all dimensions, 20.48 ≤ χ^2^s(3) ≤ 456.27, *p*s < .001 (specific test results can be found in Table S3 in Supplementary Materials).

**Table 3:** Median and Mean Scores of the Extent to Which Treatment Options Are Expected to Meet Expectations on Each Dimension

Dimension	Self-help book		Digital intervention		Blended counseling		Face-to-face counseling	
	*Mdn*	*M* (*SD*)	*Mdn*	*M* (*SD*)	*Mdn*	*M* (*SD*)	*Mdn*	*M* (*SD*)
Helps with the problem	5	4.38 (1.492)	5	5.00 (1.348)	6	6.01 (1.017)	7	6.47 (0.732)
Includes personal support	2	2.85 (1.692)	5	4.63 (1.573)	6	6.05 (1.084)	7	6.41 (0.930)
Accessible at convenient location	6	5.63 (1.466)	6	5.78 (1.282)	6	5.62 (1.211)	5	5.25 (1.331)
Accessible at appropriate time	7	6.11 (1.331)	6	5.85 (1.216)	6	5.54 (1.187)	5	4.64 (1.627)
Can be consulted anonymously	7	6.12 (1.306)	6	5.69 (1.437)	4	4.08 (1.815)	4	3.57 (1.930)
Free of charge	4	3.47 (1.736)	5	5.12 (1.481)	4	3.94 (1.541)	3	3.36 (1.725)
Motivates to get better	5	4.73 (1.537)	5	4.88 (1.425)	6	5.92 (1.027)	6	6.33 (0.788)
Connects to own learning preferences	4	4.04 (1.631)	5	4.63 (1.524)	6	5.40 (1.237)	6	5.82 (1.062)
Provides feedback	2	2.87 (1.746)	5	5.00 (1.372)	6	5.82 (1.077)	6	6.09 (0.969)
Attractive/ appeals to me	4	4.03 (1.849)	5	4.44 (1.602)	5	5.08 (1.400)	6	5.50 (1.269)
Credible	5	4.62 (1.650)	5	4.81 (1.474)	6	5.77 (1.156)	6	6.33 (0.765)
Accessible without waiting	7	6.21 (1.352)	6	5.75 (1.316)	5	5.05 (1.450)	4	3.98 (1.868)

Several dimensions relate to the content of the intervention, namely helping with the problem, motivating to get better, including personal support, providing feedback, connecting to one’s own learning preferences, attractiveness, and credibility. Pairwise comparisons were significant for helpfulness, where face-to-face was rated highest, followed by blended interventions, digital interventions, and self-help books. For personal support, face-to-face and blended interventions were rated similarly and higher than digital interventions. Self-help books were again rated least favorably. Pairwise comparisons for the motivational nature of the interventions showed the highest ratings for face-to-face, followed by blended interventions, with digital interventions and self-help books sharing the third rank. Face-to-face interventions connected most to learning preferences, followed by blended interventions, digital interventions, and self-help books (all pairwise comparisons were significant). In terms of including feedback, face-to-face and blended interventions were rated similarly and higher than digital interventions, followed by self-help books. In terms of appeal, face-to-face and blended interventions shared the first rank, followed by digital interventions and self-help books sharing the second rank. The most credible intervention option is face-to-face, followed by blended interventions, and finally digital interventions and self-help books (which shared the third rank).

Other dimensions are linked to implementation and practical considerations, specifically waiting time, accessibility at an appropriate time or location, anonymous consultation, and perceived affordability. Pairwise comparisons for the convenient location dimension showed higher ratings for self-help books and digital interventions compared to face-to-face counseling. All pairwise comparisons, apart from blended counseling vs. digital interventions, were significant for the convenient time dimension and showed the best ratings for self-help books, followed by both digital and blended interventions, with the lowest rating for face-to-face interventions. For anonymity, both self-help books and digital interventions were rated highest (and did not differ significantly from one another), followed by blended and face-to-face interventions (which also did not differ significantly). In terms of being free of charge, pairwise comparisons showed the highest ratings for digital interventions, followed by blended interventions, and finally self-help books and face-to-face interventions (the latter two did not differ significantly). Pairwise comparisons for waiting time were all significant and indicated the following order: self-help books, digital interventions, blended interventions, and face-to-face interventions.

Taken together, face-to-face counseling and self-help books generally showed opposite patterns in terms of favorable dimensions. Face-to-face counseling met the participants’ needs (i.e., was rated higher than 4) regarding helpfulness, motivation, credibility, including feedback, personal support, convenient location, appeal, and learning preferences. For affordability, anonymity, and (to a lesser extent) waiting time expectations were not met. Self-help books were rated very favorably on anonymity, waiting time, accessibility at a convenient time and location. However, they showed the least favorable ratings for many other dimensions and did not meet the needs for feedback, personal support, and being free of charge. Similar to face-to-face counseling, blended counseling met the participants’ needs for helpfulness, motivation, credibility, including feedback, personal support, convenient location, appeal, and learning preferences (although face-to-face counseling was rated higher for helpfulness, motivation, learning, and credibility). The scores for waiting time, convenient time, and affordability were better for blended counseling as compared to face-to-face counseling. Digital interventions were also rated positively on these latter criteria, but had a lower score than blended or face-to-face interventions for helpfulness, motivation, credibility, including feedback, personal support, appeal, and learning preferences.

### Self-Reported Likelihood of Use

**Figure 2:**
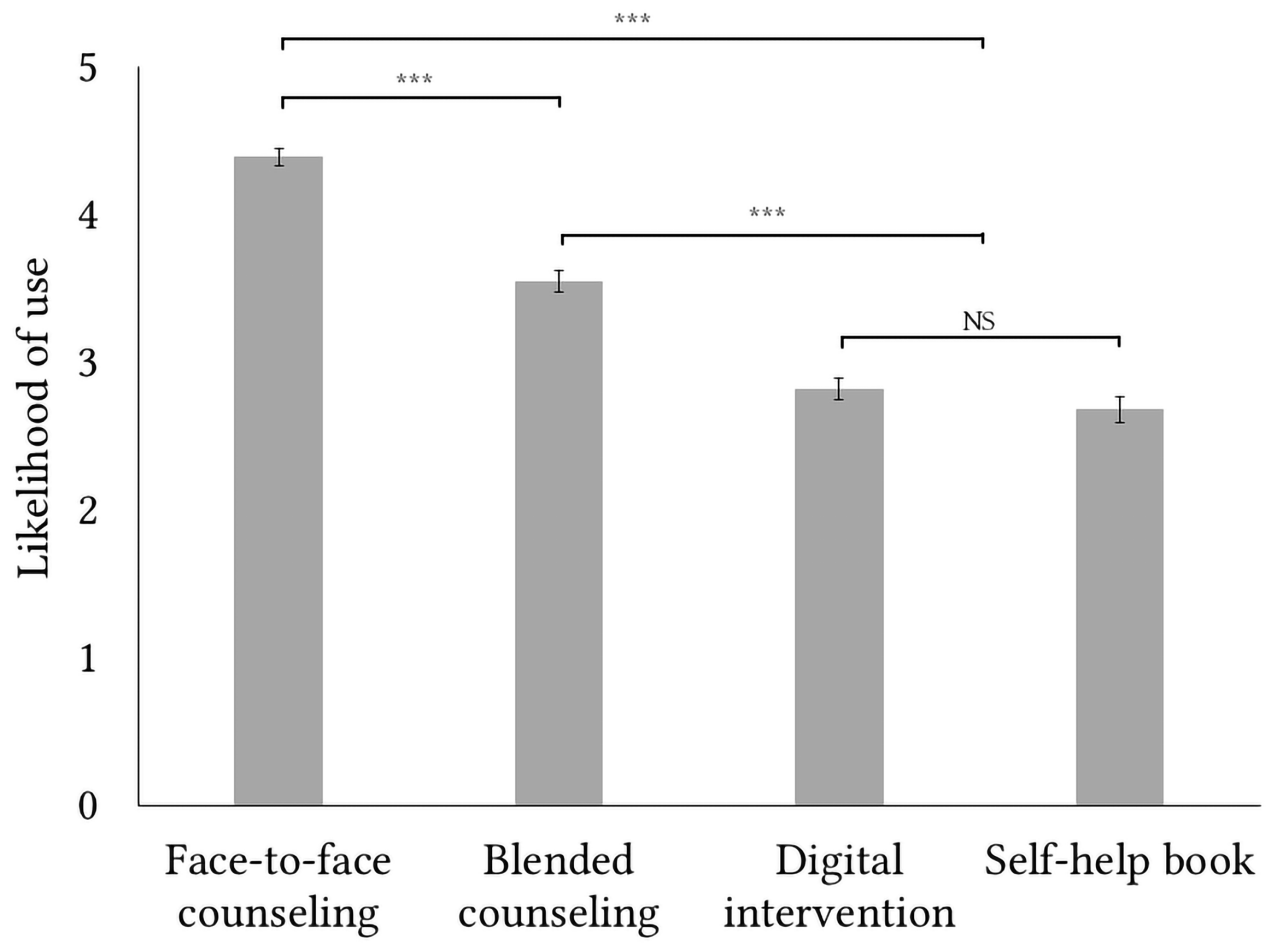
Likelihood of Using an Intervention Modality on a Scale of 0 (Very Unlikely) to 5 (Very Likely)

There were significant differences in self-reported likelihood of use between the intervention modalities, χ^2^(3) < 239.55, *p* < .001. Pairwise comparisons showed that participants were most likely to consider face-to-face counseling, followed by blended counseling and a shared third rank for digital interventions and self-help books (Figure 2). Exploration of the data did suggest substantial individual differences in self-reported likelihood of use for the intervention modalities (see also Table S4 in Supplementary Materials).

When comparing individuals with current or past mental health problems (*n* = 152) with individuals without lived experience (*n* = 63), a couple of differences stand out. A larger proportion of individuals with lived experience indicate a higher likelihood of using face to face counseling (*Mdn* = 5; *M* = 4.54, *SD* = 0.76) as compared to those without lived experience (*Mdn* = 4; *M* = 4.16, *SD* = 0.90), *U* = 3535.00, *p* < .001. For digital interventions on the other hand, individuals with lived experience indicate a lower likelihood of use (*Mdn* = 3; *M* = 2.71; *SD* = 1.18) as compared to individuals without lived experience (*Mdn* = 3; *M* = 3.13; *SD* = 1.10), *U* = 5772, *p* = .01. There are no significant differences for self-help books, *U* = 4516.00, *p* = .50, or blended counseling based on lived experience, *U* = 5172.00, *p* = .32.

## Discussion

Digital interventions are supported by a growing evidence base and can increasingly become part of accessible and personalized mental healthcare services. It is important to understand how individuals view different intervention options and what their preferences are.

The findings of Musiat et al. in 2014 indicated that face-to-face interventions were expected to perform well in terms of helpfulness, personal support, credibility, motivational aspects, suiting learning preferences, including feedback, appeal, and credibility. Books, websites, and apps performed well in terms of convenient location and time, anonymity, and no waiting time but were rated poorly on all other dimensions. This also resulted in the highest self-reported likelihood of using face-to-face interventions, followed by self-help books and web-based interventions. Smartphone applications showed the lowest likelihood of use. The current study also found relevant differences between face-to-face counseling, blended counseling, digital interventions, and self-help books across twelve evaluation dimensions. Participants indicated that they were most likely to use face-to-face interventions, followed by blended counseling. The results showed that digital and on-paper self-help options were not preferred by the participants.

The ratings of the twelve evaluation dimensions for mental health interventions showed that helpfulness, personal support, motivation and credibility were deemed very important for intervention selection. The importance of helpfulness aligns with the concept of performance expectancy in the UTAUT model, which has been identified as the strongest predictor of technology use (Venkatesh et al., 2003). Being free of charge showed to be least important according to the participants. Overall, the importance of these dimensions resembles the findings of Musiat et al. (2014). A discrete choice experiment, which forces respondents to make trade-offs, could provide further insights regarding the importance of dimensions. Phillips et al. (2021) already implemented such a design with six attributes (introductory training, human contact, peer support, proven effectiveness, mode of content delivery, and costs) in Germany. In line with our study, they also observed that personal contact with a psychotherapist in blended care and proven effectiveness were highly valued by participants. However, in contrast to our findings, low price was also deemed important.

These dimensions inform on what potential users experience as strengths and limitations of a certain modality and can therefore help researchers, developers and professionals in improving intervention design and enabling informed decision making. To give an example, digital interventions and books do not meet the needs regarding credibility, which implies that implementation can benefit from promoting system credibility in clients, e.g. by relying on the principles of persuasive theory (Fogg, 2002). Strategies (e.g., for raising awareness) should be designed to facilitate informed decision making, ideally participatively with the target groups based on needs and preferences. For instance, Vomhof et al. (2024) showed that medical students found that timing of information (early in their studies) was the most important attribute of an information strategy (more than recommendations or media channels).

Other dimensions might also be relevant for intervention design and predicting intervention acceptance. Pretorius et al. (2019) have suggested that a sense of control over a help-seeking journey and self-reliance might be a motivation for online help-seeking. Sense of control might therefore be an additional dimension that could determine preferences for intervention modality and could be interesting for further research. Stigma or the fear for reactions from others could also influence the help-seeking process (Rowe et al., 2014). Digital health literacy and mental health literacy, as well as previous (online or real-life) help-seeking behavior and experience, can also impact intervention selection. For blended interventions, clinician’s attitudes towards new interventions and technologies will also influence patient attitudes and subsequent use (Cohen et al., 2024). Future research should assess which dimensions most strongly predict likelihood of use and actual use of different intervention modalities.

Also similar to Musiat et al. (2014), there was a clear difference in which dimensions were rated positively for face-to-face counseling and self-help books. Face-to-face counseling was evaluated very favorably for the dimensions linked to the content of the intervention (e.g., helpfulness, motivation, credibility and personal support), but showed practical limitations (i.e. cost, anonymity and waiting time). On the other hand, self-help books were rated most favorably for many practical circumstances but showed the least favorable ratings for many other dimensions. As compared to self-help books, digital interventions were expected to be significantly more helpful, to include more personal support, to connect to one’s learning preferences better, and to include more feedback. This differs from the original study, where digital self-help options (apps and websites) were rated more similar to self-help books. It also differs from De Jesús-Romero et al. (2022) who compared willingness to use self-help books and smartphone applications and found a small preference for self-help books. The study suggested that this preference was influenced by participant education level, perceived availability, and perceived helpfulness of the intervention.

A new category consisted of blended counseling, which was rated fairly similar to face-to-face counseling although self-reported likelihood of use was significantly higher for face-to-face as compared to blended counseling. Blended counseling received a better rating than face-to-face counseling for waiting time, convenient time, and affordability. However, face-to-face counseling outperformed blended counseling in three top-rated dimensions, i.e., it was expected to be more helpful, motivational, credible, which could contribute to a high likelihood of use. The relevance of and preference for face-to-face contact was also documented in a general population (Phillips et al., 2021) and student sample (Kählke et al., 2024) in Germany and could be linked to the way participants are used to receiving mental healthcare (in Europe) and a potential resulting (lack of) concrete understanding of blended care. Additionally, some external factors could contribute to a lack of (intended) use of technology-enhanced mental healthcare, as shown by Butz et al. (2022) who point to factors such as technical or reimbursement issues.

A substantial number of respondents experienced current (29%) or past (36%) mental health problems. Exploratory analyses showed that these individuals indicated a higher likelihood of using face-to-face counseling as compared to those without lived experience (although only one individual had actual experience with this modality). Individuals with lived experience indicated a lower likelihood of using digital interventions than those without mental health problems. This appears to contrast with the findings in the review by Borghouts et al. (2021) which found higher willingness to use digital mental health when symptoms were more severe. However, this review also observed that certain mental health symptoms (e.g., depressive symptoms) can reduce motivation or ability to interact with interventions and severe symptoms can impede actual intervention engagement (Borghouts et al., 2021).

To be able to compare views regarding different delivery modalities, we needed to make abstractions from specific interventions and provide a general description of what a certain modality could look like. Therefore, opinions and preferences were formulated in the context of a (rather abstract) mental health delivery modality while acceptance is not a unitary concept. When presenting more concrete interventions (and according intensities and target populations), other preferences might arise. The specific wording used in the descriptions could also impact credibility and other ratings. The current study did not differentiate between guided and unguided digital interventions. Other studies which provided more concrete and tailored information about blended interventions have obtained more encouraging findings regarding the acceptability of blended treatment for first-episode psychosis (Valentine et al., 2020) or depression and anxiety (Braun et al., 2023). It is a limitation that the current study consisted of a sample which is quite young and has very little experience with mental health services despite the relatively large number of individuals with current or past mental health problems. The study combined students and individuals from the general population and we observed differences in gender and tablet use, which is in line with statistics on technology use in Flanders (Northern Belgium) showing elevated tablet use in adults as compared to emerging adults (De Marez et al., 2025). The current study gathered data on the self-reported likelihood of using a certain modality, in line with the ‘intention to use’ concept as framed in theories such as the UTAUT or the Technology Acceptance Model (TAM; Venkatesh et al., 2003). While it is an extensively investigated construct (e.g., Wei et al., 2024), a gap between intention and behavior has been documented in these theories. Measuring actual use of (self-help) services in a general sample is challenging and requires a longitudinal panel study. Further adding to the complexity of assessing this concept, mere exposure to information in a questionnaire could already result in changed behavior, e.g., registration for mental health programs (Apolinário-Hagen et al., 2023). Factors contributing to intervention selection were rated using Likert scales, which is an approach relying heavily on self-reflection while participants might not always have full insight into one's own decision-making processes. Future research can benefit from going beyond the questionnaire format and could implement (1) qualitative data collection to collect more in-depth information about why certain dimensions are deemed important or why certain modalities are preferred; or (2) discrete choice experiments based on the current and potential additional intervention dimensions, which force respondents to make trade-offs between different features and are less sensitive to bias (e.g., based on social desirability and lacking experience). Investigating perceptions of more concrete interventions (e.g., a guided self-help website for depression) or new technologies (e.g., chatbots) could also be interesting ways to extend the current work.

### Conclusion

Taken together, the current study suggests that mere self-help (online or on paper) is not expected to sufficiently meet the needs and is not the preferred choice for handling mental health problems for most individuals. However, participants did show positive views towards digital resources, and a subgroup would likely use the digital and self-help options. If presented with the choice, individuals still prefer face-to-face counseling. Nevertheless, our and related work identify blended interventions as a promising treatment option for the future. Informing patients about the available options, underlying mechanisms, and potential added values – a practice shown to increase acceptance in the past (Ebert et al., 2019; Linardon et al., 2022) – remains a meaningful strategy, as does tailoring interventions to patient preferences and needs (Van Daele et al., 2020). While the evidence base and technology for digital mental health services is largely there, it is important to document patient expectations and resulting acceptance.

## Supplementary Materials

The Supplementary Materials contain additional graphs supporting the findings in the publication, including sample demographics and test results (see De Witte et al., 2025S).



De WitteN. A. J.
BuelensF.
Apolinário-HagenJ.
Van DaeleT.
 (2025S). Supplementary materials to "Attitudes and expectations towards mental health interventions in the general population: Comparing face-to-face counseling, blended counseling, and digital or on-paper self-help"
[Additional information]. PsychOpen. 10.23668/psycharchives.21309
PMC1292317741725698

## Data Availability

The data that support the findings of this study are available from the corresponding author, [NDW], upon reasonable request.

## Preregistration

The study was not preregistered.

## Reporting Guidelines

This article is written according to the JARS-QUANT guidelines.

## Social Media Accounts

*Nele A. J. De Witte:* LinkedIn, *Fien Buelens:* LinkedIn, *Jennifer Apolinário-Hagen:* LinkedIn, *Tom Van Daele:* LinkedIn
